# Patterns of Change in Cognitive Function over Six Months in Adults with Chronic Heart Failure

**DOI:** 10.1155/2012/631075

**Published:** 2012-08-15

**Authors:** Barbara Riegel, Christopher S. Lee, Dale Glaser, Stephen T. Moelter

**Affiliations:** ^1^School of Nursing, University of Pennsylvania, 418 Curie Boulevard, Philadelphia, PA 19104-4217, USA; ^2^School of Nursing Portland Campus, 3455 SW US Veterans Hospital Road, SN-6N, Portland, OR 97239, USA; ^3^Glaser Consulting, San Diego, CA, USA; ^4^University of the Sciences in Philadelphia, Behavioral and Social Sciences, 217 Kline Hall, 600 S. 43rd St, Philadelphia, PA 19104, USA

## Abstract

Few investigators have studied cognition over time in adults with heart failure (HF). A battery of neuropsychological tests was administered to 279 adults with chronic systolic or diastolic HF at baseline, three and six months. Growth mixture modeling (GMM) was used to model the measure anticipated to be most sensitive, the digit symbol substitution task (DSST). We describe how and why the DSST patterns change over time. Other measures of cognition were examined to identify consistency with the DSST patterns. The sample was predominantly male (63.2%), Caucasian (62.7%), mean age 62 years. The best fit GMM revealed two trajectories of DSST scores: *Average* processing speed group (40.5%) and *Below Average* processing speed (59.9%). Neither group changed significantly over the six month study. Other measures of cognition were consistent with the DSST patterns. Factors significantly associated with increased odds of being in the *Below Average* processing speed group included older age, male gender, Non-Caucasian race, less education, higher ejection fraction, high comorbid burden, excessive daytime sleepiness, and higher BMI. As some of the factors related to cognitive impairment are modifiable, research is needed to identify interventions to preserve and improve cognition in these patients.

## 1. Introduction

The prevalence of cognitive impairment in adults with chronic heart failure (HF) is recognized as a factor contributing to complexity in caring for these patients [1]. A review of studies from 2002 to 2007 estimated that approximately 25–50% of HF patients have impaired cognition [2]. Few investigators have studied if and how cognition changes over time in this population, however. Thus, the purposes of this study were to identify patterns of cognition and how those patterns change over a six-month period in adults with chronic HF and to identify contributors to the patterns identified.

 Only six studies were located in which investigators measured cognitive function of HF patients over intervals of 18 weeks [3] to 10 years [4]. Two of the six studies enrolled hospitalized HF patients [5, 6]. Three of the six assessed cognitive functioning using screening instruments such as the Mini-Mental Status Examination (MMSE) or the Hodkinson Abbreviated Mental Test (AMT) rather than standardized neuropsychological measures [5–7]. Only one of these three screening studies found a change in functioning over time [5]. That study was a retrospective analysis of data from 1,220 elderly hospitalized HF patients from 81 clinical sites in Italy collected during the 1990s. Cognitive functioning was screened with the AMT before hospital discharge when an ACE inhibitor was initiated for some patients. After 12 months, ACE inhibitor prescription was associated with significantly improved cognitive functioning (odds ratio, 1.57; 95% confidence interval, 1.18–2.08), after adjusting for baseline cognitive functioning and potential confounding variables including systolic blood pressure. Cognitive functioning did not improve among patients without HF.

 Three of the six longitudinal studies used neuropsychological measures of cognition. One of these studies demonstrated that global cognitive functioning on the Dementia Rating Scale (DRS) improved significantly, but very modestly, over a 12-month period in patients with HF compared to those with cardiovascular disease but not HF [8]. Specifically, patients with HF improved in attention, initiation/perseveration, and conceptualization over time. The only predictor of improved global cognitive function in the HF patients over time was higher diastolic blood pressure at baseline (*r* = 0.38; *P* = .02). Perhaps the most important finding from that study was to show that cognitive functioning in HF is most likely to either increase slightly or remain stable over a 12-month interval. 

The second study using neuropsychological tests measured cognitive functioning in response to an exercise training program [3]. Twenty HF patients (NYHA class III) who completed an 18-week exercise training program were compared to five HF patients who were unable to participate in training. Six measures of cognitive functioning were administered at baseline and at completion of the program. Patients who exercised had significantly improved scores in attention and psychomotor speed.

In the final study, data from 702 octogenarians from the Swedish Twin Registry were analyzed using latent growth curve modeling to assess performance over time in persons with HF compared to those without HF [4]. Cognitive testing was done five times between 1991 and 2002 using a battery of neuropsychological measures of processing speed, visuospatial ability, and short-term, episodic, and semantic memory. Measures of episodic memory declined more over time in the 13% of the sample with HF compared to those without HF. There were no significant differences between the groups in the other cognitive domains.

Together these studies suggest that episodic memory declines over time but attention, initiation/perseveration, conceptualization, and psychomotor speed may improve over time in some HF patients. Factors associated with improved cognition included receipt of an ACE inhibitor and exercise training. The effect of blood pressure on cognitive change is unclear. Because data that address change in cognition is so sparse and inconsistent, we analyzed data obtained from a sample of adults with chronic HF in whom a battery of neuropsychological measures was administered at baseline, three and six months. This analysis began with cognitive processing speed as measured by the Digit Symbol Substitution Test (DSST) [9]. Previous research has shown that DSST is among the most sensitive cognitive measures to a variety of cognitive disturbances and declines rapidly with advancing age [10]. Lower DSST scores were shown to be associated with increased five-year mortality in the Cardiovascular Health Study [11] and reduced information processing speed using a similar measure (Symbol Digit Modalities) is associated with cerebrovascular pathology and cardiovascular risk [12, 13].

We used the DSST as an initial measure that we believed would be highly sensitive to any cognitive impairment if it was present. But, we were also interested in describing the differing neurocognitive patterns associated with the latent groups established by DSST performance. It was possible, though not highly likely, that our well-functioning group on the DSST would show a more impaired profile in another neurocognitive domain or that we would show different patterns of cognitive test performance in other measures. Thus, DSST, with measures at three time points, was selected as the primary study endpoint to assess longitudinal change. But change in the other measures is described as well.

## 2. Methods

The methods used in this study have been described previously but, in brief, we conducted a prospective cohort study with a consecutive sample of 280 adults with chronic HF [14]. Subjects were enrolled from three outpatient settings in the northeastern U.S. Only adults with chronic Stage C HF (currently or previously symptomatic) of either systolic or diastolic type were enrolled. All willing participants were screened to assure adequacy of visual acuity, hearing, and English fluency. We excluded individuals with severe cognitive impairment as measured by the Telephone Interview of Cognitive Status (TICS) [15]; nine individuals with a TICS score below 24 were ineligible for enrollment. Thereafter, we divided the sample into those with and without mild cognitive impairment. The primary analysis focused on the effect of excessive daytime sleepiness on self-care, so individuals living in a long-term care setting, working nights or rotating shifts, or noted to have severe renal failure requiring dialysis, major depressive illness, an imminently terminal illness, plans to move out of the area, or a history of serious drug or alcohol abuse within the past year were excluded. Most of the data were collected in person during home visits. Clinical information was abstracted from the medical record. Data were collected between 2007 and 2010. 

### 2.1. Measurement

A neuropsychological test battery measuring the cognitive domains of processing speed, simple and complex attention, working memory, verbal memory, and crystallized cognitive ability was administered to all participants at enrollment, three and six months. This six-month interval was chosen to facilitate comparison with our prior research and with the realization that a significant proportion of the HF population dies in the first year after diagnosis [16]. The battery included the Digit Symbol Substitution Test [9] (DSST), which measures processing and psychomotor speed, the Psychomotor Vigilance Task (simple attention) [17], the Trail Making Test B (complex attention) [18], the Probed Recall Memory Task (working memory) [10], and the Letter Number Sequencing test (short-term memory) [9]. 

As noted above, the DSST was the focus of this analysis. To complete the DSST, subjects were provided a series of nonsequential numbers with blank boxes below. A key at the top shows each unique number paired with a different symbol. Working in order, participants transcribe the appropriate symbol for each number in the blank boxes. Speed is enhanced if the subject recalls the symbol that goes with each letter rather than needing to look at the key. The number correctly completed in 120 seconds is measured. Higher scores on the DSST indicate better cognitive processing speed. Age-specific norms are available but in this study the raw score was used in analysis because the analytic technique requires continuous scores. Instead of using age-specific norms, we included age as a covariate in the analysis of determinants of cognitive processing speed.

Sociodemographic characteristics of age and gender were measured by self-report. Household income was self-reported in terms of adequacy; participants were asked to consider how well their household lives on its income (comfortable, have more than enough to make ends meet; have enough to make ends meet; do not have enough to make ends meet). Premorbid (i.e., crystallized) intelligence was measured with the American National Adult Reading Test (ANART) as a proxy for formal intelligence testing [19]. On the ANART, subjects read a list of 50 phonetically irregular words aloud. To estimate intelligence, we calculated the number of errors made on the ANART and applied the formulas [19].

To determine exercise frequency at enrollment, participants were asked how much exercise they had obtained in the past week (none or fewer than 30 minutes, <1 hour (minimal), or 1–3 hours (adequate)). We asked about only the past week to avoid issues with recall. Body mass index was calculated from height and weight in pounds at the time of enrollment using the standard formula. 

Blood pressure was measured during the enrollment home visit and used to compute mean arterial pressure. NYHA functional class was rated by a single cardiologist using information from a structured interview [20]; higher classes indicate worse functional status. Last known left ventricular ejection fraction was abstracted from the medical record. ACE inhibitor and beta blocker usage was assessed (yes or no) from the list of medications obtained from patients at the baseline visit. The 19-item Charlson Comorbidity Index was used in analysis [21]. Responses are weighted and summed. The total or categories (low, moderate, or high) can be used in analysis, but we used the categories because in the original validity testing the instrument authors demonstrated that comorbidity categories predicted mortality, complications, health care resource use, length of hospital stay, discharge disposition, and cost [22]. 

Daytime sleepiness was measured with the Epworth Sleepiness Scale (ESS); values greater ≥ 11 indicate excessive daytime sleepiness [23]. Respondents rate the likelihood of falling asleep in eight soporific situations using a 4-point Likert scale ranging from never dozing (0) to high chance of dozing (3). The ESS correlates significantly with the frequency of apneas and is a validated research tool in the assessment of excessive daytime sleepiness [24]. Test-retest reliability (*r* = 0.82) and internal consistency (*α* = 0.88) have been established in addition to its single factor structure [25]. 

Medication adherence was assessed with the Basel Assessment of Adherence Scale (BAASIS), a structured interview assessing general medication adherence over the past month [26]. The BAASIS is a 4-item tool assessing compliance with taking and timing dimensions of medication regimens as well as the occurrence of drug holidays over the past four weeks. A positive answer on any of the questions classifies a patient as nonadherent with the medication regimen, a strict definition of nonadherence that increases the sensitivity of measurement. The BAASIS has established reliability and validity [26]. 

### 2.2. Analysis

Standard descriptive statistics of frequency, central tendency, and dispersion were used to describe the sample. Comparisons of characteristics between observed trajectories were made using Student's *t*-tests, without assuming equal variance, or *χ* ^2^ analysis where appropriate. StataMP v11 (College Station, Texas) was used for all descriptive and comparative statistics. There is a growing body of literature on effectively capturing heterogeneous trajectories of change over time [27–29]. Finite mixture models of longitudinal data, such as growth mixture modeling (GMM), are used to identify common and distinct trajectories of change [30]. GMM was used in this study to permit intra- and inter-individual variability [31]. 

Mplus 6.1 software [32] was used to generate GMM, using the expectation maximization (EM) algorithm and full information maximum likelihood (FIML) for missing data, assuming that data were missing at random (MAR) [33]. A maximum of three latent class trajectories was posited, based on theoretical expectations and the limited sample size. Our approach to model specification in GMM was based on procedures explicated by Ram and colleagues [34]. Model fit was assessed with Akaike information criterion (AIC); the model with the lowest value was favored. Given the potential sensitivity of the AIC to sample size and model complexity, entropy also was used to gauge the accuracy of classification. Entropy ranges from 0 to 1 with values closer to 1 indicating better trajectory separation based on posterior probabilities (average probability of belonging in “most likely” trajectory near 1.0) [35, 36]. Model selection was also assessed using the Lo-Mendell-Rubin adjusted likelihood ratio test [LRT] with a significant result indicating the *k* (as opposed to the *k* − 1) class solution is preferred [37]. Finally, the proportion of the sample in each trajectory (not less than 5%) and parametric bootstrap LRT were used to compare alternative models [38]. Univariate (unadjusted) and multivariate (adjusted) logistic regression modeling was then used to quantify the relationship between baseline demographic and clinical characteristics and the likelihood of membership in the trajectory with the lowest scores. 

## 3. Results

One subject was missing data on the key variables, leaving a sample of 279 available for this analysis. The sample was predominantly male (63.2%) and Caucasian (62.7%) (Table 1). The mean age of the participants was 62 years. Family income was sufficient in a large proportion (48.8%), and the average education was approximately 14 years. Overall ANART error scores suggest average general intelligence (estimated full scale IQ (FSIQ) *T* Score = 47, 39-40th percentile). Most participants were functionally compromised (76.3% NYHA class III or IV). A slight majority (53.1%) had low-comorbid burden based on Charlson Comorbidity Index categories. Many were sedentary (20.4%) or exercised less than 1 hour weekly (36.9%). The average BMI was in the overweight (25–29) range. Approximately 24% of the sample had significant excessive daytime sleepiness. 

**Figure 1:**
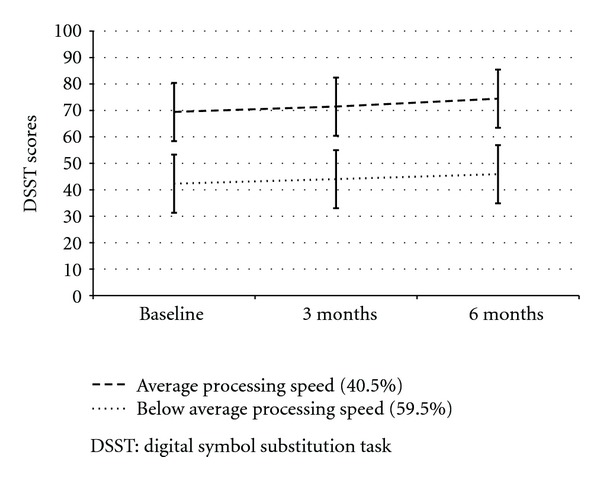
Two trajectories of change over time in DSST scores.

Over the course of the study, average DSST scores improved minimally from 53.4 ± 17.5 at baseline to 55.8 ± 17.6 at three months and 58.1 ± 17.9 at six months, and DSST scores were highly correlated over time (*r* > .83, *P* < .001). The most informative and best fit GMM revealed two trajectories of change in DSST scores (Figure 1). Model AIC was 6112.5, entropy was 0.808, and the Lo-Mendell-Rubin adjusted LRT (326.9, *P* < .001) and parametric bootstrap LRT (346.2, *P* < .001) indicated dominant fit with two trajectories over considering a single pattern of change in DSST scores. The smaller trajectory (40.5% of the sample, average posterior probability = 94.6%) identified participants who had higher DSST scores throughout the study, referred to as the *average* processing speed group. The ANART estimated FSIQ for this group was in the average range (*T* = 49, 44-45th percentile). The larger trajectory (59.9% of the sample, average posterior probability = 94.1%) identified participants who had lower DSST scores throughout the study, referred to as the *below average* processing speed group. This group was also in the average range of general intelligence on the ANART (*T* = 46, 34-35th percentile). 

There were several unadjusted differences between the two observed trajectories (Table 1). For example, patients in the *average* processing speed group were younger, had more years of education, a lower mean arterial pressure, lower BMI, and a slightly better, albeit not clinically meaningfully different, ANART score compared with patients in the *below average* processing speed group. A greater proportion of patients in the *average* processing speed group were female, Caucasian, had sufficient or greater income, had adequate exercise, had a low level of comorbidity, and took an ACE inhibitor compared with patients in the *below average* processing speed group. 

In addition to the DSST, all other measures of cognition were different between the two groups at baseline. Each measure demonstrated a very minor improvement in each group over time. Differences in each measure of cognition remained significant between the two groups at three and six months (Table 2).

There were several factors that increased the likelihood that patients would fit the *below average* processing speed group individually and in the multivariate model (Table 3); multivariate model *χ* ^2^ = 139.9, *P* < .001; McFadden's pseudo *R* ^2^ = 37.3%; Hosmer-Lemeshow LRT = 255.5, p.533; correct classification rate = 79.1%; area under the curve = .876. Factors significantly associated with increased odds of being classified in the *below average* processing speed group in the multivariate model included additional years of age, male gender, non-Caucasian race, fewer years of education, higher ejection fraction, high comorbid burden, excessive daytime sleepiness, and higher values of BMI. Factors not related to the *below Average* group were income, exercise, blood pressure, ACE inhibitors, beta blockers, NYHA functional class, premorbid intelligence, and medication adherence.

## 4. Discussion

 Two distinct patterns of cognitive performance were identified in this sample of functionally limited but relatively young HF patients. The *below average* processing speed group scored consistently and significantly lower than the *average* processing speed group on every measure of cognition, in spite of evidence of average premorbid intellect in both groups. The major finding of this study was that there was little change in cognition over the six months of the study in either of these groups. The minimal improvement seen probably reflects the learning that typically occurs when people take these tests multiple times. Many of the predictors of those in the *below average* processing speed group were not modifiable (higher age, male gender, non-Caucasian race, fewer years of education, and high comorbid burden). However, excessive daytime sleepiness and BMI predicted group membership and these factors are potentially modifiable, as discussed below.

Other investigators have found some improvement in cognition over time, although we did not. The study by Zuccalá and colleagues described in the introduction enrolled hospitalized HF patients, treated them with ACE inhibitors, and documented an improvement in overall cognitive functioning after 12-months [5]. Our results could differ from theirs because we enrolled out-patients, most were already on an ACE inhibitor, and we followed them for only 6 months. The other studies that demonstrated improvement in cognition tested an exercise intervention [3] or followed patients for a longer period of time [8]. At this point it does not appear that cognition can be greatly improved but further research is needed to clarify this issue.

Building on the results of previous investigators we tested the influence of ACE inhibitors, exercise, and blood pressure on cognitive change over time and although these three factors were significantly different between the groups in unadjusted analyses, none of these factors were useful in predicting *below average* processing speed. Our results probably differ from those of prior investigators because of the analytic technique used. That is, had we simply compared the two groups our results would have supported those of prior investigators. However, because we used a regression analysis to identify the best predictors of *below average* processing speed, some of the predictors were supplanted by other better predictors. 

The results of this study provide some support for the conceptual model published by Bennett and colleagues [39]. They proposed that age, gender, comorbidity, hypertension, depression, medications, education, and circulatory insufficiency (i.e., ejection fraction, duration of HF, NYHA functional status, and oxygen saturation) contribute to cognitive deficits in adults with chronic HF. Our results corroborate their model with age, gender, comorbidity, education, and ejection fraction identified as significantly associated with increased odds of being classified in the *below average* processing speed group. Noting that a high Charlson Comorbidity score at enrollment predicted *below average* processing speed suggests that processing speed is influenced by illness burden, which supports prior results [11, 40]. These results differs from those of Pressler et al. [41] who found that comorbidity was not associated with cognitive changes, which may reflect the manner in which the Charlson Comorbidity Index score was used in their analysis (continuous score versus our use of categories) and the longitudinal nature of our analyses. 

We found that fewer years of education predicted *below average* processing speed. These results support those of Stern et al. who reported incident dementia data from a follow-up study of 593 community-based, nondemented individuals aged 60 years or older and identified low education as a risk factor for dementia [42]. Stern discusses these findings in terms of cognitive reserve or individual differences in the cognitive processes or neural networks underlying task performance that allow some people to cope better than others with damage to the brain [43]. 

Excessive daytime sleepiness and BMI may be targets of intervention to improve cognition. Excessive daytime sleepiness may be a reflection of poor sleep quality and/or sleep-disordered breathing, which are known to accentuate the often subtle cognitive problems existing in persons with HF [44, 45]. Sleep quality can be improved with pharmaceutical agents, exercise [46], and cognitive behavior therapy for insomnia [47]. Sleep-disordered breathing is treatable with continuous positive airway pressure, which has the added benefit of improving ejection fraction [48]. Body mass index has not been studied as a predictor of cognition previously in adults with HF, but higher BMI has been found to be negatively associated with cognition in other groups [49, 50]. 

A surprising finding of our study was that higher ejection fraction was associated with *below average* processing speed, a finding that contradicts those of others who have shown that lower ejection fraction is associated with impaired cognition [51–53]. It may be that an unaccounted for interaction between ejection fraction and another factor such as a medication could have influenced the direction of effect in this study. It should be noted, however, that in some studies no relationship was identified between ejection fraction and cognition [54, 55]. Further research is needed to clarify the relationship between ejection fraction and cognition.

Limitations of this analysis include the relatively young age of the sample, considering that HF is most prevalent in the eighth decade [16], adequate income, and an average education of 14 years—all characteristics that suggest that results from this sample may not be generalizable to the general HF population. It is important to note that the parent study used a cohort design in which individuals with mild cognitive decline were preferentially sampled, which may explain the two trajectories identified here. Also, as these were out-patients, there was no guarantee that the body weight reflected adipose or body mass rather than fluid accumulation. Strengths of this study include the longitudinal data, a relatively large sample size, and the sophisticated analytic technique. 

Future research is needed describing how cognition changes or fails to change over time and what modifiable factors are associated with an improvement. Clearly impaired cognition is an issue in these patients, but we remain uncertain what causes it, whether it is temporary or permanent, and how to intervene. Further research examining the relationship between obesity and cognition is needed in HF patients. Higher inflammatory markers are known to increase brain and heart injury; cognition research including biomarkers would further our understanding of how body weight affects cognition.

In summary, close to half of our sample demonstrated *average* processing speed but most of the sample demonstrated *below average* processing speed and no improvement in cognition was seen over the six months of this study. Efforts are needed to identify interventions that can effectively prevent and perhaps reverse cognitive decline after HF is diagnosed. Right now, exercise is the most promising approach, as it has been demonstrated to induce an increase in neural precursor cell activity and improve cognition directly [56]. Exercise also could influence the two modifiable predictors identified in this study: BMI and excessive daytime sleepiness. Research testing interventions aimed at improving cognition are urgently needed.

## Figures and Tables

**Table 1:** Clinical and demographic characteristics of the two groups. Mean ± standard deviation or *n* (%) is reported.

	Total sample	*Average* processing speed (*n* = 114)	*Below a* *ve* *ra* *ge* processing speed (*n* = 165)	*P* value
Age (years)	62.1 ± 12.4	56.1 ± 12.1	66.3 ± 11.9	<.001
Male	179 (64.2)	62 (54.4)	117 (70.9)	.005
Race/ethnicity				
White	175 (62.7)	86 (75.4)	89 (53.9)	<.001
Non white	104 (37.3)	28 (24.6)	76 (46.1)	
Income				.021
More than enough	98 (35.1)	43 (37.7)	55 (33.3)	
Sufficient	136 (48.8)	61 (53.5)	75 (45.5)	
Less than enough	45 (16.1)	10 (8.8)	35 (21.2)	
Years of education	13.9 ± 2.9	14.9 ± 3.1	13.2 ± 2.5	<.001
Body mass index (BMI)	31.0 ± 7.9	29.4 ± 7.5	32.0 ± 8.1	.006
Exercise				.002
None	57 (20.4)	19 (16.7)	38 (23.0)	
Minimal	103 (36.9)	32 (28.1)	71 (43.0)	
Adequate	119 (42.7)	63 (55.3)	56 (34.0)	
Charlson comorbidity severity				<.001
Low	148 (53.1)	79 (69.3)	69 (41.8)	
Moderate	101 (36.2)	32 (28.1)	69 (41.8)	
High	30 (10.7)	3 (2.6)	27 (16.4)	
NYHA functional class				
Classes I and II	66 (23.7)	33 (28.9)	33 (20.0)	.188
Class III	163 (58.4)	63 (55.3)	100 (60.6)	
Class IV	50 (17.9)	18 (15.8)	32 (19.4)	
Ejection fraction	35.5 ± 17.0	34.5 ± 16.0	36.2 ± 17.6	.404
ACE inhibitor	161 (57.7)	75 (65.8)	86 (52.1)	.023
Beta blocker	258 (92.5)	107 (93.9)	151 (91.5)	.466
Mean arterial pressure	84.7 ± 12.0	82.6 ± 11.4	86.1 ± 12.2	.016
Epworth sleepiness scale score ≥ 11	66 (23.7)	23 (20.2)	43 (26.1)	.256
Basel medication adherence scale	0.96 ± 0.94	1.10 ± 0.95	0.90 ± 0.94	.193
ANART correct responses	56.8 ± 1.4	57.3 ± 1.3	56.4 ± 1.3	<.001
Digit symbol substitution task total score	53.4 ± 17.5	69.4 ± 11.0	42.3 ± 11.5	<.001

ACE: angiotensin converting enzyme; ANART: American National Adult Reading Test; NYHA: New York Heart Association.

**Table 2:** Change in indices of cognition by group. Mean ± standard deviation is reported.

Measure of cognition	Enrollment	3 months	6 months	*F*-test^†^
Digit symbol substitution task total score				
*Adequate *processing speed	69.4 ± 11.1	71.5 ± 11.7	74.5 ± 10.7	*F* = 421.7^‡^
*Below average* processing speed	42.3 ± 11.5	44.0 ± 11.0	45.9 ± 11.1	
Psychomotor vigilance task lapses				
*Adequate *processing speed	3.8 ± 2.6	3.4 ± 2.8	3.5 ± 2.5	*F* = 23.9^‡^
*Below average* processing speed	5.8 ± 3.8	5.4 ± 3.4	5.1 ± 3.4	
Trail making Test B time				
*Adequate *processing speed	77.1 ± 32.8	74.6 ± 32.8	67.4 ± 31.2	*F* = 88.8^‡^
*Below average* processing speed	134.6 ± 61.7	128.7 ± 57.3	127.4 ± 57.5	
Probed recall memory task (out of 4)				
*Adequate *processing speed	2.6 ± 1.2	2.7 ± 1.2	2.9 ± 1.2	*F* = 45.5^‡^
*Below average* processing speed	1.6 ± 1.2	1.9 ± 1.3	2.0 ± 1.3	
Letter number sequencing test				
*Adequate *processing speed	10.4 ± 3.2	10.5 ± 3.4	10.8 ± 3.7	*F* = 51.0^‡^
Below *average* processing speed	7.5 ± 3.2	7.7 ± 3.1	7.7 ± 3.2	
Number of cognitive tests with impairment				
*Adequate *processing speed	1.3 ± 0.7	1.4 ± 0.8	1.3 ± 0.8	*F* = 29.8^‡^
*Below * *average* processing speed	2.0 ± 1.1	1.9 ± 1.1	1.7 ± 1.0	

^†^Test of between-subjects effects; ^‡^ *P*  value < .0001.

**Table 3:** Factors predicting *below average* processing speed: unadjusted and adjusted odds.

	Unadjusted odds ratio (95% CI)	*P* value	Adjusted odds ratio (95% CI)	*P* value
Age	1.08 (1.06–1.11)	<.001	1.12 (1.08–1.16)	<.001
Female	0.48 (0.30–0.81)	.005	0.42 (0.21–0.85)	.016
Non-Caucasian	2.62 (1.55–4.43)	<.001	2.82 (1.21–6.55)	.016
ANART-FSIQ	0.61 (0.50–0.74)	<.001	0.75 (0.54–1.03)	.078
Ejection fraction	1.01 (0.99–1.02)	.410	1.02 (1.00–1.05)	.033
Income	1.43 (1.01–2.04)	.046	1.41 (0.83–2.41)	.208
Charlson moderate^∗^	2.47 (1.45–4.19)	.001	1.81 (0.90–3.64)	.096
Charlson high^∗^	10.30 (2.99–35.45)	<.001	9.65 (1.77–52.54)	.009
ACE inhibitor	0.57 (0.35–0.93)	.024	0.67 (0.23–1.34)	.259
Beta blocker	0.71 (0.28–1.81)	.467	1.55 (0.38–6.41)	.543
Years of education	0.80 (0.73–0.88)	<.001	0.85 (0.74–0.98)	.024
Mean arterial pressure	0.99 (0.99–1.00)	.296	0.99 (0.99–1.01)	.179
NYHA	1.38 (0.99–1.92)	.056	0.85 (0.52–1.38)	.506
Excessive daytime sleepiness	1.39 (0.79–2.48)	.257	2.42 (1.05–5.53)	.037
BMI	1.05 (1.01–1.08)	.007	1.06 (1.01–1.11)	.009
Basel adherence score	0.84 (0.65–1.09)	.192	1.06 (0.61–1.22)	.402
Exercise minimal^†^	1.11 (0.56–2.21)	.768	0.92 (0.35–2.44)	.865
Exercise adequate^†^	0.44 (0.23–0.86)	.016	0.61 (0.24–1.58)	.313

^∗^Relative to low Charlson comorbid category.

^†^Relative to no exercise.

Key: ANART: American National Adult Reading Test; ACE Inhibitor: angiotensin converting enzyme inhibitor; NYHA: New York Heart Association.
